# In vivo estimation of elastic heterogeneity in an infarcted human heart

**DOI:** 10.1007/s10237-018-1028-5

**Published:** 2018-05-17

**Authors:** Gabriel Balaban, Henrik Finsberg, Simon Funke, Trine F. Håland, Einar Hopp, Joakim Sundnes, Samuel Wall, Marie E. Rognes

**Affiliations:** 1 Division of Imaging Sciences and Biomedical Engineering, King’s College London, St. Thomas Hospital, London, UK; 2 Simula Research Laboratory, Oslo, Norway; 3 Department of Informatics, University of Oslo, Oslo, Norway; 4 Department of Radiology and Nuclear Medicine, Oslo University Hospital, Rikshospitalet, Oslo, Norway; 5 Department of Mathematical Science and Technology, Norwegian University of Life Sciences, Ås, Norway; 6 Department of Cardiology, Center for Cardiological, Oslo University Hospital, Rikhospitalet, Oslo, Norway

**Keywords:** Adjoint method, Data assimilation, Cardiac mechanics, Myocardial infarction, Elastography

## Abstract

In myocardial infarction, muscle tissue of the heart is damaged as a result of ceased or severely impaired blood flow. Survivors have an increased risk of further complications, possibly leading to heart failure. Material properties play an important role in determining post-infarction outcome. Due to spatial variation in scarring, material properties can be expected to vary throughout the tissue of a heart after an infarction. In this study we propose a data assimilation technique that can efficiently estimate heterogeneous elastic material properties in a personalized model of cardiac mechanics. The proposed data assimilation is tested on a clinical dataset consisting of regional left ventricular strains and in vivo pressures during atrial systole from a human with a myocardial infarction. Good matches to regional strains are obtained, and simulated equi-biaxial tests are carried out to demonstrate regional heterogeneities in stress–strain relationships. A synthetic data test shows a good match of estimated versus ground truth material parameter fields in the presence of no to low levels of noise. This study is the first to apply adjoint-based data assimilation to the important problem of estimating cardiac elastic heterogeneities in 3-D from medical images.

## Introduction

Myocardial infarction (MI) is a condition in which muscle tissue in the heart is damaged due to a loss of blood supply. After an infarction, there is an increased risk of further complications, such as rupture, infarct expansion, ventricular remodelling, hypertrophy, and heart failure (Holmes et al. 2005). Post-MI, the elastic properties of the myocardium have been shown to play a large role in determining the outcome (Morita et al. 2011; Fomovsky et al. 2012).

A promising way to study the elastic properties of in vivo myocardium is by mathematical modelling and computer simulation. With simulation it is possible to create an in silico representation of a patient’s heart after an infarction. This opens up new possibilities for quantification of elasticity, beyond what is available in medical imaging today. Additionally, an in silico model that is personalized to a patient can potentially simulate the effects of treatments or therapies on the patient, thereby improving the outcome and reducing risks after MI.

Previous studies have estimated elasticity as a global value in models of infarcted hearts (Chabiniok et al. 2009; Gao et al. 2014; Fan et al. 2016). This resulted in simulated pressure–volume relations that matched well to those observed in vivo. Additionally, estimated elasticity values were shown to be significantly higher in patients with infarction compared to healthy controls (Fan et al. 2016). While these results are intriguing, the use of global parameters neglects the fact that infarction is a local phenomenon.

A more detailed approach has been to identify infarcted and healthy regions a priori and then define separate parameters for these regions in a model (Walker et al. 2005; Mojsejenko et al. 2015; McGarvey et al. 2015). The resulting regional parameters were shown to be higher in the infarcted region as compared to the healthy remote myocardium. This demonstrates the potential of modelling to quantify differences in tissue stiffness within the same heart. However, the infarctions that caused the stiffness differences were induced in otherwise healthy animals, leading to clearly demarcated regions of myocardial infarction. In the general clinical setting, however, patients may suffer from multiple infarctions, possibly occurring at different times and locations, and/or may be suffering from other cardiac pathologies. Such conditions may lead to substantial heterogeneity in elastic properties, not known a priori.

To address the issue of spatial heterogeneity in cardiac elasticity, we here present a novel 3-D data assimilation procedure. This procedure employs an adjoint gradient-based optimization method which can efficiently handle high-dimensional parameter sets. In turn, this allows for the spatial resolution of heterogeneous elastic parameters throughout the myocardium. Previous studies on the topic of soft tissue elastography have proposed the adjoint gradient approach with 2-D models and synthetic data (Oberai et al. 2003) for both compressible (Gokhale et al. 2008) and incompressible mechanics (Goenezen et al. 2011). Furthermore, we applied adjoint gradient optimization to the problem of estimating local cardiac contraction (Balaban et al. 2017; Finsberg et al. 2017), but did not consider spatially resolved elastic parameters, which we now address.

We demonstrate the utility of our method by personalizing an in silico model of cardiac elasticity to data collected from a patient in heart failure with a previous myocardial infarction and a heterogeneous distribution of fibrotic tissue. Input data consist of regional strains, which are computed by speckle tracking echocardiography, and a pressure transient obtained from a catheter. Additionally, we quantify the patient’s cardiac scar burden from late gadolinium enhanced magnetic resonance images (LG-MRI) to provide a context for the modelling results.

## Methods and materials

### Clinical data

Clinical data were obtained with the permission of the Oslo University Hospital in the context of the Impact study (Hospital 2016). Specifically, we consider the case of a 64-year-old man in systolic heart failure, with left bundle branch block, coronary artery disease, and chronic infarction predominantly in the inferior and inferolateral sections of the left ventricular wall.

Prior to treatment, the patient had echocardiography, LG-MRI, and left ventricular (LV) pressure measurements taken, which are the basis for the clinical data used in this study. Pressure recordings were carried out with an intra-vascular pressure sensor catheter (Millar micro catheter: precision 1 mmHg, accuracy 1.5 mmHg Millar 2017); that is, a pressure catheter that was positioned in the LV via the right femoral artery. Pressure data were obtained automatically and digitized (Powerlab system, AD Instruments) before offline analyses were performed with a low-pass filter of 10Hz.

**Fig. 1:**
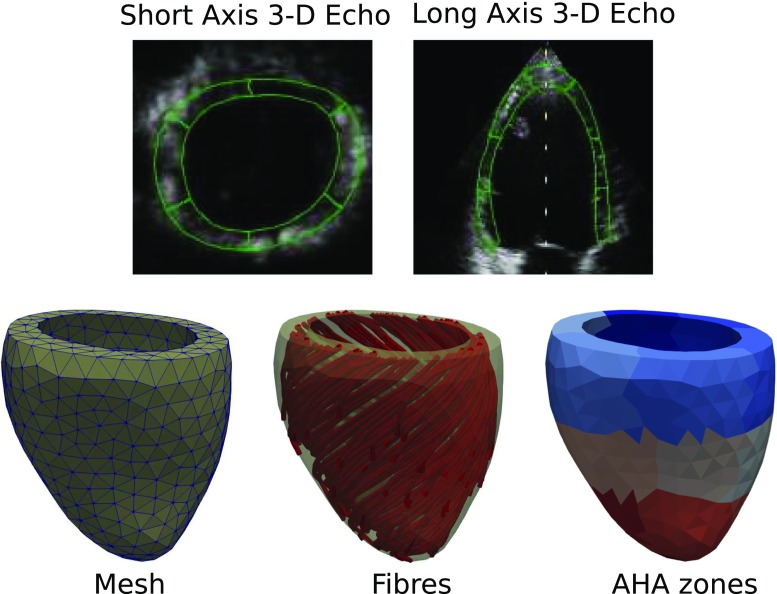
Top row: Example short- and long-axis slices taken from 3-D echocardiography with tracked segments in green. Bottom row: Model LV geometry derived from the 3-D echo data. From left to right are the computational mesh, rule-based fibre orientations and the standard AHA zones shown in separate colours

A 4-D echocardiography examination of the patient’s LV was performed using a GE Vingmed E9 machine. Speckle tracking motion analysis was carried out with GE’s software package EchoPac. Data from 6 heartbeats were combined in order to obtain a single sequence of images for a single heartbeat. Example short- and long-axis slices taken from the image sequence are shown in Fig. 1. Seven separate measurement points of left ventricular strain during atrial systole were obtained from the echo images. The strains were given as regional averages defined for a standard 17 segment AHA representation (Cerqueira et al. 2002) and measured in the local longitudinal, radial, and circumferential directions, without any off-diagonal shear components.

Strain and pressure data were synchronized using begin of atrial systole (BAS) as the first point of registration. In the pressure data, BAS was located by a deflection in a simultaneously acquired left atrial electrogram. In the strain data, BAS was identified by the onset of longitudinal stretching following diastasis. Pressures corresponding to strains were registered by the use of image acquisition times until just before ventricular systole, which was identified in the strain data by the onset of longitudinal contraction.

**Fig. 2:**
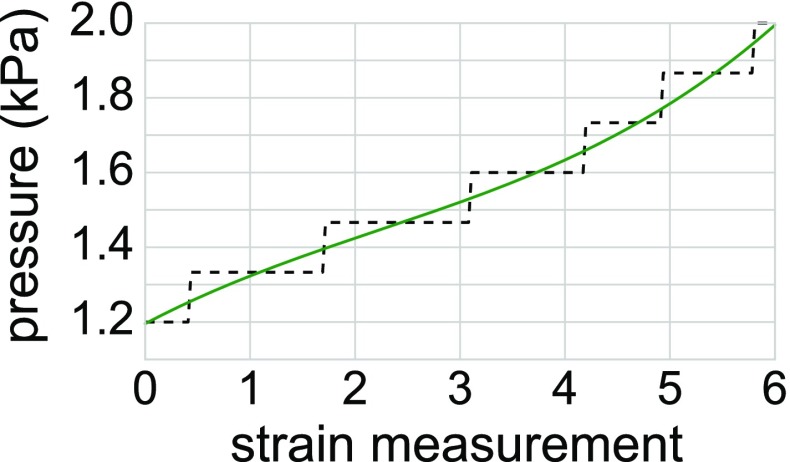
Left ventricular pressure trace synchronized to echo-derived strain measurements taken in atrial systole. The original catheter data are shown in dotted black, whereas the cubic polynomial smoothed data are shown in solid green

Pressure increases in late diastole are generally very small in magnitude, and for our patient strain points 2 and 3 shared the same pressure measurement. In order to give each strain point a unique pressure, an additional cubic polynomial smoothing was carried out. Both smoothed and original pressure data are illustrated in Fig. 2.

**Fig. 3:**
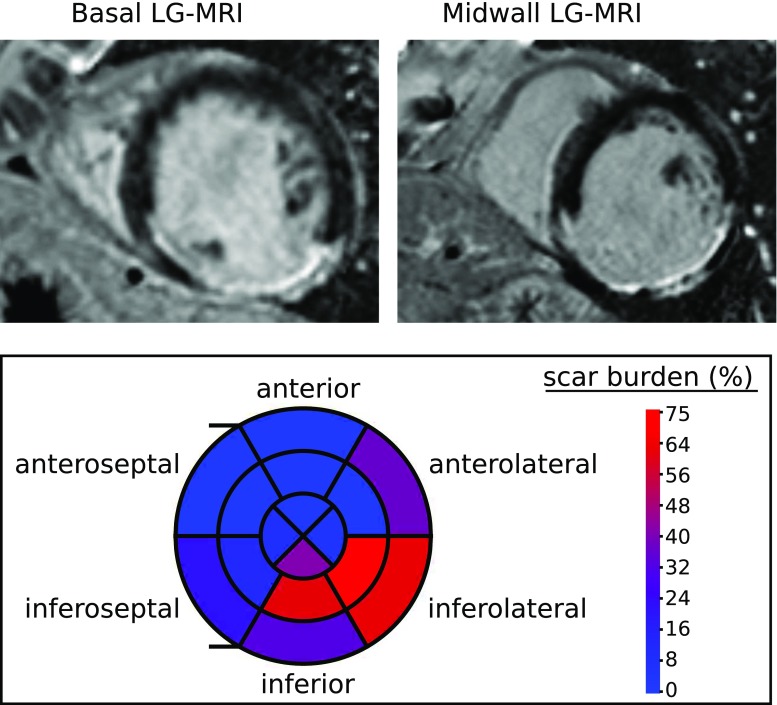
Top row: two example short-axis late enhancement gadolinium MRI images used for regional scar quantification. Fibrotic sections of the myocardium appear in white. Bottom row: regional quantification of myocardial scar burden based on LG-MRI. The inner, middle, and outer rings represent apical, midwall, and basal sections, respectively. The RV insertion points are marked by two horizontal lines extending to the left of the bullseye

Cardiac magnetic resonance imaging was performed with a 3.0 Tesla scanner (Skyra, Siemens, Erlangen, Germany). We quantified the amount of myocardial fibrosis on a per region basis from short-axis late gadolinium enhancement images acquired 10–20 min after intravenous injection of 0.2 mmol/kg of gadoterate meglumine (Guerbet, Villepinte, France). This resulted in an estimated volume ratio of fibrotic to healthy tissue for each myocardial segment (scar burden). In this analysis the apex region was merged into the neighbouring apical regions, giving a 16 segment division. Example LG-MRI images and the scar burden data are displayed in Fig. 3.

### Mesh and fibre generation

We created a computational mesh based on a 3-D ultrasound image to capture the details of the patient ventricular geometry in an in silico model. The image was taken at the start of atrial systole, when the pressure was at a minimum. Using GE’s EchoPac software, we extracted triangulated data points for the left ventricular endocardial and epicardial surfaces. These surfaces were cut by a plane fitted to the basal points of the surfaces, and adjusted so that the ventricular volume of the computational mesh was within 1 mL of the volume measured in the image. Using the epicardial, endocardial, and basal surfaces as boundaries, we created a volumetric mesh using Gmsh (Geuzaine and Remacle 2009). This mesh contained 741 vertices and 2214 tetrahedra. AHA zones were delineated on this volumetric mesh based on data provided by EchoPac, so that our AHA zones were consistent with those used to calculate image-based strains.

Local myocardial fibre orientations were assigned with a helix angle of 40 degrees on the endocardium rotated clockwise throughout the ventricular wall to $-50$ degrees on the epicardium using a rule-based method (Bayer et al. 2012). Snapshots of the image-based geometry, along with AHA segments and fibres, are shown in the bottom row of Fig. 1.

### Elastic wall motion model

We adopt a quasi-static continuum mechanics framework to simulate the motion of the left ventricle throughout atrial systole. As primary variables, we consider a vector field $\mathbf {u}$ giving the displacement map between a reference configuration $\varOmega$ and a deformed configuration undergoing a pressure load. Furthermore, we define the deformation gradient $\mathbf {F}= {{\mathrm{Grad}}}\mathbf {u}+ \mathbf{I}$.

In our wall motion model the myocardium is considered to be a hyperelastic material with strain energy given by a transversely isotropic simplification of the Holzapfel–Ogden law (Holzapfel and Ogden 2009),

1 $$\begin{aligned} \psi ( \mathbf C ) = \frac{a}{2 b} \left( e^{b (I_1( \mathbf C ) - 3)} -1 \right) + \frac{a_f}{2b_f} \left( e^{ b_f (I_{4f}( \mathbf C ) - 1)_+^2} -1 \right) . \end{aligned}$$

The energy density $\psi$ in (1) defines the amount of elastic energy stored per unit volume myocardium, given the values of the right Cauchy–Green tensor $\mathbf C = \mathbf {F}^{T} \mathbf {F}$. The notation $(\cdot )_{+}$ refers to $\max \{\cdot , 0\}$, and the mechanical invariants $I_1$ and $I_{4f}$ are defined as

2 $$\begin{aligned} I_1( \mathbf C ) = {{\mathrm{tr}}} \mathbf C , \quad \quad I_{4f} = \mathbf {e_f}\cdot \mathbf C \, \mathbf {e_f}, \end{aligned}$$

with $\mathbf {e_f}$ indicating the local myocardial fibre direction field.

The material parameters $a, a_f, b, b_f$ are scalar-valued quantities which influence the stiffness of the material. We allow these material parameters to vary spatially with a piecewise linear representation, so that each material parameter has a separate value at each vertex of the mesh. For the sake of improved numerical stability (Land et al. 2015), we employ a modified strain energy density $\tilde{\psi }$ in place of $\psi$ with

3 $$\begin{aligned} \tilde{\psi }( \mathbf C ) = \psi \left( J^{-\frac{2}{3}} \mathbf C \right) , \end{aligned}$$

where $J = \det \mathbf {F}$ is the deformation gradient. The elastic energy (3) is embedded into a standard pressure–displacement variational formulation of incompressible hyperelasticity [Chapter 8.5 of Holzapfel (2000)]. Displacements are set to 0 in the longitudinal direction at the base of the ventricular geometry by a Dirichlet boundary condition. Movement in the other directions at the base is restricted by a linear spring with constant $k = 1.0 \text { kPa}$ as in our previous study (Balaban et al. 2017).

The total variational equation, including the effects of blood pressure, $p_{\text {blood}}$, and the basal spring, is given by: find the displacement $\mathbf {u}$ and the hydrostatic pressure *p* such that

4 $$\begin{aligned} \begin{aligned}&\int _{\varOmega } \left( \mathbf {P}+ p J \mathbf {F}^{-T} \right) : {{\mathrm{Grad}}}\delta \mathbf {u}\, \mathrm {d}V+ \int _{\varOmega } (J - 1) \delta p \, \mathrm {d}V\\&\quad + \int _{\partial \varOmega _{\text {base}}} k \, \mathbf {u}\cdot \delta \mathbf {u}\, \mathrm {d}S+ p_{\text {blood}}\int _{\partial \varOmega _{\text {endo}}} J \mathbf {F}^{-T} \mathbf{N}\cdot \delta \mathbf {u}\, \mathrm {d}S= 0, \end{aligned} \end{aligned}$$

for all admissible variations $\delta \mathbf {u}, \delta p$ in the displacement and pressure respectively. In (4), $\mathbf {P}$ is the first Piola–Kirchhoff tensor: $\mathbf {P}= \frac{\partial \tilde{\psi }}{\partial \mathbf {F}}$, $\partial \varOmega _{\text {endo}}$ represents the endocardium and $\partial \varOmega _{\text {base}}$ the ventricular base, and $\mathbf{N}$ is the unit outward facing boundary normal. We discretize (4) by a mixed finite element method with Taylor–Hood interpolation (Hood and Taylor 1974); that is, a piecewise quadratic representation of the displacement field and a piecewise linear representation of the pressure.

The software implementation of the finite element vector and matrix assembly code is based on the software package FEniCS  (Logg et al. 2011). Nonlinear systems are solved using the PETSc SNES implementation of a Newton line search algorithm (Balay et al. 2015), while the inner linear solves are handled by a distributed memory parallel LU solver (Li and Demmel 2003).

### Elastic parameter estimation via constrained minimization and adjoint gradient calculations

We consider a least squares minimization of the mismatch between model derived and measured strains, to personalize the elastic material properties of our computational mechanics model.

We compute both model and measured strains in terms of the deformation gradient tensor $\mathbf {F}$ and multiply the model strains by $\mathbf {F}_0^{-1}$, which is the strain at the smallest measured in vivo pressure. This allows for the simulated strains to be calculated from a reference that is at the same pressure as that used for the image-based strains (Nikou et al. 2016). For a given echo image number *i*, AHA region $\varOmega _j$, and strain direction *k*, we compute the model strain as

5 $$\begin{aligned} \mathbf {F}_\mathrm{model}^{i,j,k} = \frac{1}{|\varOmega _j|} \int _{\varOmega _j} \mathbf{e}_k \cdot \mathbf {F}_i \mathbf {F}_0^{-1} \mathbf{e}_k \, \mathrm {d}V \end{aligned}$$

where $|\varOmega _j|$ is the AHA segment volume, and $\mathbf{e}_k$ the unit vector pointing in the direction *k*.

The image-based strain measurements are given as regional engineering strains, which we relate to a diagonal component of the deformation gradient by

6 $$\begin{aligned} \mathbf {F}_\mathrm{measured}^{i,j,k} = \varepsilon ^{i,j,k} + 1 \end{aligned}$$

where $\varepsilon$ is the engineering strain and $\mathbf {F}_\mathrm{measured}$ the corresponding measured deformation gradient diagonal component. We note that this implies the linear approximation $\varepsilon _k \approx \nabla \mathbf {u}\cdot \mathbf{e}_k$.

We quantify the mismatch between model and measured strains with the following functional

7 $$\begin{aligned} I_{\text {data}}= \sum _{i = 1}^{N_{m}} \sum _{j = 1}^{N_{r}} \sum _{k \in \{c,r,l\}} \left( \mathbf {F}_\mathrm{model}^{i,j,k} - \mathbf {F}_\mathrm{measured}^{i,j,k} \right) ^2. \end{aligned}$$

Here $N_{m}=7$ is the number of strain measurements available in atrial systole and $N_{r}=16$ the number of AHA regions, with the apex segment excluded for compatibility with the LG-MRI data. Finally, the direction set of index *k* refers to the circumferential (c), radial (r), or longitudinal (l) directions.

We allow each of the four elastic material parameters $a, b, a_f, b_f$ in (1) to vary in space, and more precisely, to vary as a continuous piecewise linear function defined relative to the computational mesh. This allows us to resolve spatially heterogeneous material parameters, at the cost of greatly increasing their dimensionality. To constrain the minimization problem at hand, we introduce a first-order Tikhonov regularization functional favouring more smooth material parameter sets. This regularization functional is defined as:

8 $$\begin{aligned} I_{\text {smooth}}= \frac{1}{|\varOmega |} \sum _{z \in \{a, a_f, b, b_f \}} \int _{\varOmega } | {{\mathrm{Grad}}}z |^2 \, \mathrm {d}V, \end{aligned}$$

where $|\varOmega |$ is the volume of the simulated myocardium.

In total, we consider the optimization problem of minimizing a combined data and smoothness functional over the admissible material parameter fields $a, b, a_f, b_f$:

9 $$\begin{aligned} \min _{a, b, a_f, b_f} I = \min _{a, b, a_f, b_f} \left( I_{\text {data}}+ \lambda I_{\text {smooth}}\right) \end{aligned}$$

with regularization parameter $\lambda$.

The total functional (9) is minimized by simultaneously optimizing all of the degrees of freedom of the 4 elastic parameters. This optimization is carried out by a sequential quadratic programming (SQP) algorithm (Kraft 1988). Each iteration of the SQP algorithm requires one or more evaluations of the functional (9), and the gradient of the functional with respect to all of the material parameter variables. This gradient is calculated efficiently by the adjoint gradient method [Eq. 13 of Balaban et al. (2016)] symbolically derived by the software package dolfin-adjoint (Farrell et al. 2013). In particular, the computational cost of the adjoint gradient does not significantly depend on the number of optimization parameters, of which there are 2964 in our study. This compares favourably with a one sided finite difference approach to functional gradient calculation, which would require 2964 model realizations, one for each optimization parameter.

We employ a continuity scheme (Gokhale et al. 2008) to reduce the number of nonlinear solves needed to evaluate the functional (9). In this scheme the first time the functional is evaluated the cavity pressure is applied in small increments, and the displacement–pressure solutions are saved at the seven recorded in vivo pressures. For further functional evaluations, the hyperelastic equation is solved directly at the seven pressure levels, with the previously stored displacement-pressure solution as the initialization point. If convergence in the Newton solver is not achieved, then the difference between the previous and current material parameter vector is divided into smaller increments, which are then applied. In our implementation the number of divisions is doubled every time that convergence is not achieved. Using such divisions we obtained convergence in all cases in our study.

## Numerical results

The main results of this study are heterogeneous elastic material parameters optimized to match clinical data, presented in Section 3.2. We also present simulated equi-biaxial extensions tests based on regional averages of estimated elastic parameters. Prior to the presentation of the main results, we present results for a synthetic data test for the purpose of verification and inspection of algorithm performance in Section 3.1.

Optimizations were carried out until the norm of the projected gradient was less than $1.0 \times 10^{-4}$, or 500 iteration of the SQP algorithm had been reached. A lower bound of 0.4 was applied to all material parameter fields pointwise during optimization.

### Parameter estimation and evaluation using synthetic data

For the purpose of verification of the model and the optimization procedure, we consider initial trials using synthetically generated data over the ventricular mesh. In these trials, the *ground truth* elastic parameters were defined as:

10 $$\begin{aligned} \begin{aligned} a^{0} = 2 - \frac{y}{y_\mathrm{max}}, \quad a_f^{0} = 2 + \frac{y}{y_\mathrm{max}}, \\ b^{0} = 2 - \frac{z}{z_\mathrm{max}}, \quad b_f^{0} = 2 + \frac{z}{z_\mathrm{max}}, \end{aligned} \end{aligned}$$

where $y_\mathrm{max}$ and $z_\mathrm{max}$ are the maximum absolute coordinate values in the y and z directions of the computational mesh (and where the yz-plane was defined by the basal plane). Using these ground truth parameters, average regional strains were generated by solving (4) for 6 LV blood pressures: $p_\mathrm{blood} \in \{0.1, 0.2, 0.3, 0.4, 0.5, 0.6\}$ (kPa). Four sets of strains were generated: one noise-free case and three noisy cases. For the noisy cases, realizations of Gaussian noise with standard deviations of 0.1, 0.2, and 0.3 mm were applied to the displacements from which strains were calculated. We quantified the effect of this noise on the value of the synthetic strains in the second column of Table 1. We note that though the average effect of the noise is small, individual strains have relative errors as high as 24, 25, and 12 per cent for the 0.1, 0.2, and 0.3 mm noise levels, respectively.

Optimizations were carried out using the synthetic strains as target data in the total functional (9). All material parameters were initialized to a spatially constant value of 1.5. For each level of noise, the values $\lambda ~\in ~\{1, 10, 100, 1000, 10000\}$ of the regularization parameter were tested, and the case with the lowest relative $L^2$-error, averaged across the 4 parameters, was selected. The $\lambda$ values that were selected are listed in Table 1. As expected, the regularization value increases with the noise level. In the target functional (7), $\mathbf {F}_0^{-1}$ was calculated from the model strains at 0.1 kPa.

We remark that, in order to represent non-trivial material parameters, the synthetic material parameter fields were chosen with a nonzero spatial gradient. In turn, this gave a nonzero contribution from the regularization functional, cf. (8). Thus, even in the case of an exact optimization, we did not expect to obtain an optimal functional value of 0 and did not expect to recover the exact material parameter fields in this test case.

**Fig. 4:**
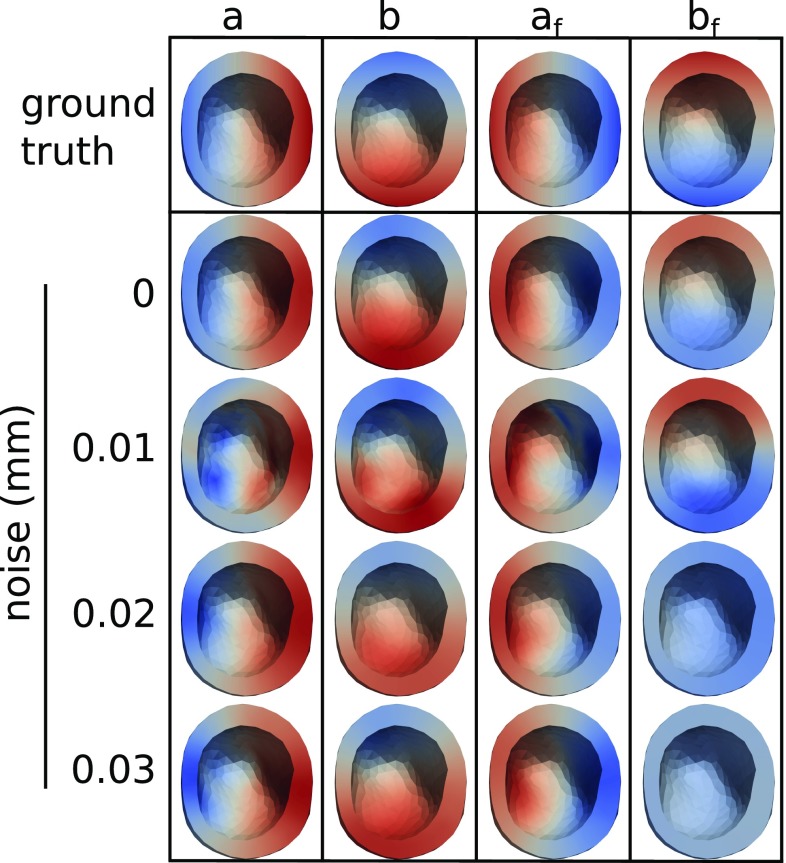
Top view of the ground truth and estimated parameters fields of the synthetic data test

The ground truth and estimated material parameter fields for this noise-free case are presented in Fig. 4. Moreover, the differences between the ground truth and estimated parameters are given in terms of the relative $L^2$-errors in Table 1, along with the optimal data and smoothness functional values.

We note that for the noise-free and 0.1 mm noise case, the parameters are accurately reproduced, with all relative errors being less that 6%. For the 0.2 and 0.3 mm noise cases, the errors in the first three material parameters *a*, *b*, $a_f$ are also less than 6%, but the error in the parameter $b_f$ is 19% and 17% for 0.2 mm and 0.3 mm noise respectively. The accuracy of the reproductions is also visible in Fig. 4, where we can see that the linear gradients are reproduced for $a, b, a_f$ in all cases and for $b_f$ in the noise-free and 0.1 mm cases.

**Table 1:** Optimal functional values and relative errors in reconstructed material parameter fields obtained in the synthetic data test

Noise (mm)	Relative strain error % mean ( % max)	$\lambda$	$I_{\text {data}}$	$I_{\text {smooth}}$	*E*(*a*)	*E*(*b*)	$E(a_f)$	$E(b_f)$
0.0	–	1	$3.4 \times 10^{-4}$	$3.3 \times 10^{-4}$	0.016	0.017	0.022	0.042
0.1	0.26 (24)	10	0.046	$9.4 \times 10^{-4}$	0.051	0.038	0.058	0.041
0.2	0.17 (25)	1000	0.33	$2.3 \times 10^{-4}$	0.029	0.060	0.038	0.19
0.3	0.36 (12)	1000	0.55	$3.3 \times 10^{-4}$	0.034	0.044	0.036	0.17

Synthetic data are corrupted by varying levels of Gaussian noise applied to the ground truth displacement field. The standard deviations of these noise realizations are given in the first column. The relative strain error due to noise is given in the second column. The numbers presented are the mean, and max of the quantities $\frac{\mathbf {F}_\mathrm{base} - \mathbf {F}_\mathrm{noisy}}{\mathbf {F}_\mathrm{base} - 1}$, calculated for each echo image, AHA zone, and strain direction. Both the baseline strain $\mathbf {F}_\mathrm{base}$ and noisy strain $\mathbf {F}_\mathrm{noisy}$ are calculated as in Eq. (5). For a parameter *c* the relative error $E(c) = \frac{\Vert c - c^{0}\Vert }{\Vert c^{0} \Vert }$, where $\Vert \cdot \Vert$ denotes the standard $L^2(\varOmega )$ norm and $c^{0}$ the ground truth parameter field

### Parameter estimation using patient-specific strain data

As a first step towards creating a patient-specific model of the infarcted left ventricle in atrial systole, we identified suitable values for the regularization parameter $\lambda$.

**Fig. 5:**
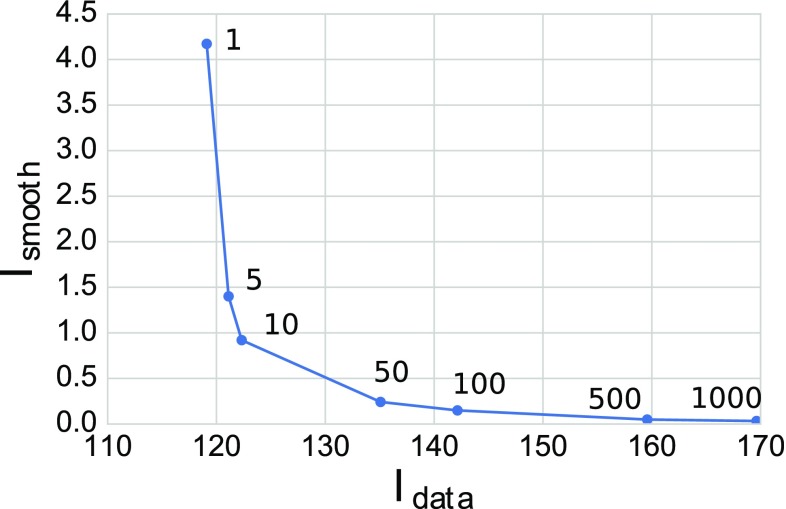
Optimal data functional value versus optimal smoothness functional value for a series of optimization experiments with clinical data over a range of regularization parameter values $\lambda$. The regularization parameter values are stated next to the corresponding data point

We tested a series of trial material parameter optimizations with the patient strains as target data using $\lambda \in \{1, 5, 10, 50, 100, 500, 1000\}$. Before optimization, material parameters were initialized with global values $a = 1.291$ kPa, $b = 5.0, a_f = 2.582$ kPa, and $b_f = 5.0$ cf. (Asner et al. 2015, Table 5, case P2). Optimal data and regularization functional values $I_{\text {data}}$ and $I_{\text {smooth}}$ were obtained for each of the $\lambda$ values tested. These are shown in Fig. 5. For the subsequent experiments, we selected $\lambda = 5$ as the corresponding optimal functional values lay in a corner of the trade-off curve, and therefore represented a good compromise between smoothness and data fit. This choice of $\lambda$ is inspired by the so called L-criterion (Hansen and O’Leary 1993).

**Table 2:** Results of parameter estimations with patient data starting from 20 points drawn from a Latin hypercube design

*I*	$I_\mathrm{data}$	$I_\mathrm{smooth}$	*a* (kPa)	*b*	$a_f$ (kPa)	$b_f$	Function evaluations
121.29	119.62	1.67	7.87 (7.09)	2.71 (4.06)	2.61 (3.34)	2.24(2.22)	514
121.51	119.97	1.54	7.63 (7.17)	2.81 (4.11)	2.51 (3.37)	2.05 (2.11)	524
121.54	119.88	1.67	8.01 (7.01)	2.67 (3.10)	2.93 (3.51)	2.52 (2.45)	583
121.62	119.99	1.62	8.22 (6.85)	2.47 (3.58)	3.83 (4.20)	1.88 (1.82)	511
121.68	120.067	1.61	8.11 (6.75)	2.49 (3.62)	3.84 (4.23)	2.25 (2.16)	450
121.81	120.28	1.53	5.80 (5.68)	2.96 (3.97)	1.64 (2.32)	3.11 (2.63)	500
121.97	120.44	1.53	8.25 (6.82)	2.65 (3.80)	2.66 (3.12)	2.92 (2.56)	501
122.76	121.28	1.48	6.40 (6.41)	2.76 (3.70)	2.23 (2.86)	1.93 (1.82)	501
124.44	122.88	1.56	6.14 (5.83)	3.06 (4.07)	2.94 (3.38)	3.77 (3.51)	501
125.05	123.54	1.51	5.92 (5.82)	2.84 (3.73)	1.80 (2.34)	4.20 (3.68)	502
125.21	123.65	1.56	6.50 (5.10)	2.94 (3.99)	3.81 (3.55)	1.95 (1.00)	501
126.42	125.02	1.40	6.15 (4.76)	3.15 (4.04)	1.60 (1.90)	2.15 (1.84)	501
127.93	126.33	1.60	5.58 (4.32)	3.24 (4.05)	1.51 (1.97)	4.56 (3.42)	501
128.83	127.64	1.19	3.98 (3.94)	3.33 (3.97)	0.88 (0.85)	2.83 (2.32)	501
129.08	127.61	1.47	5.04 (4.52)	3.09 (3.83)	2.24 (2.58)	4.80 (3.91)	501
130.07	128.39	1.68	4.59 (4.28)	3.37 (4.05)	5.67 (4.36)	1.77 (2.08)	501
131.33	129.83	1.50	6.08 (4.14)	2.87 (3.74)	3.27 (3.08)	4.55 (3.49)	501
138.35	136.50	1.85	3.44 (3.17)	3.79 (4.02)	4.47 (2.74)	2.11 (2.23)	501
170.72	169.43	1.29	3.09 (2.33)	3.93 (3.57)	6.00 (2.17)	1.44 (1.27)	501
171.14	170.51	0.63	2.66 (1.55)	2.82 (2.57)	1.26 (0.81)	2.81 (1.69)	501

Optimized material parameter fields are given as a spatial mean followed by the standard deviation in brackets. Standard deviations are calculated as the $L^2(\varOmega )$ distance of a parameter field to its mean, normalized by the mesh volume. The first three columns show the value of the optimized total, data, and smoothness functionals. The last column shows the number of least squares function evaluations needed for convergence. A limit of 500 SQP iterations was used, with each SQP iteration consisting of 1 or more function evaluations

With the value of the regularization parameter $\lambda$ fixed, we carried out a series of optimizations using various initializations for the elastic parameters. These initializations consisted of 20 global parameter sets whose values were taken from a Latin hypercube design (McKay et al. 1979) with minimum and maximum limits of 0 and 10 respectively for each variable. This design created parameters which spanned the parameter space with low redundancy. Optimal functional values for the optimizations are shown in Table 2 along with the spatial mean and standard deviation for each elastic parameter. We note that there is great variability in the optimal parameter sets calculated, and that there is a clear best fitting parameter set. Furthermore, the values of the smoothness functional are similar among all parameter sets and small in comparison to the total functional values. This indicates that the parameter sets differ in their ability to fit the model to the data, but are similar in their smoothness.

**Fig. 6:**
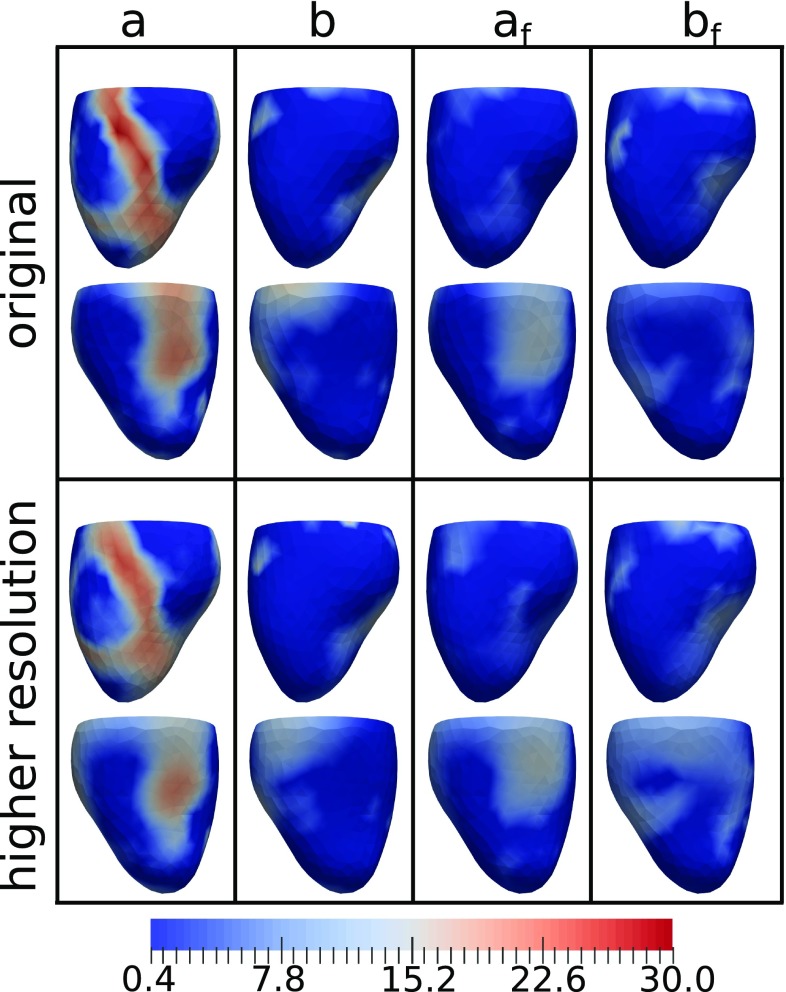
View of optimal material parameters at two different mesh resolutions estimated from patient strain data. The first and third rows show the inferior view and the second and fourth rows the anterior view

**Fig. 7:**
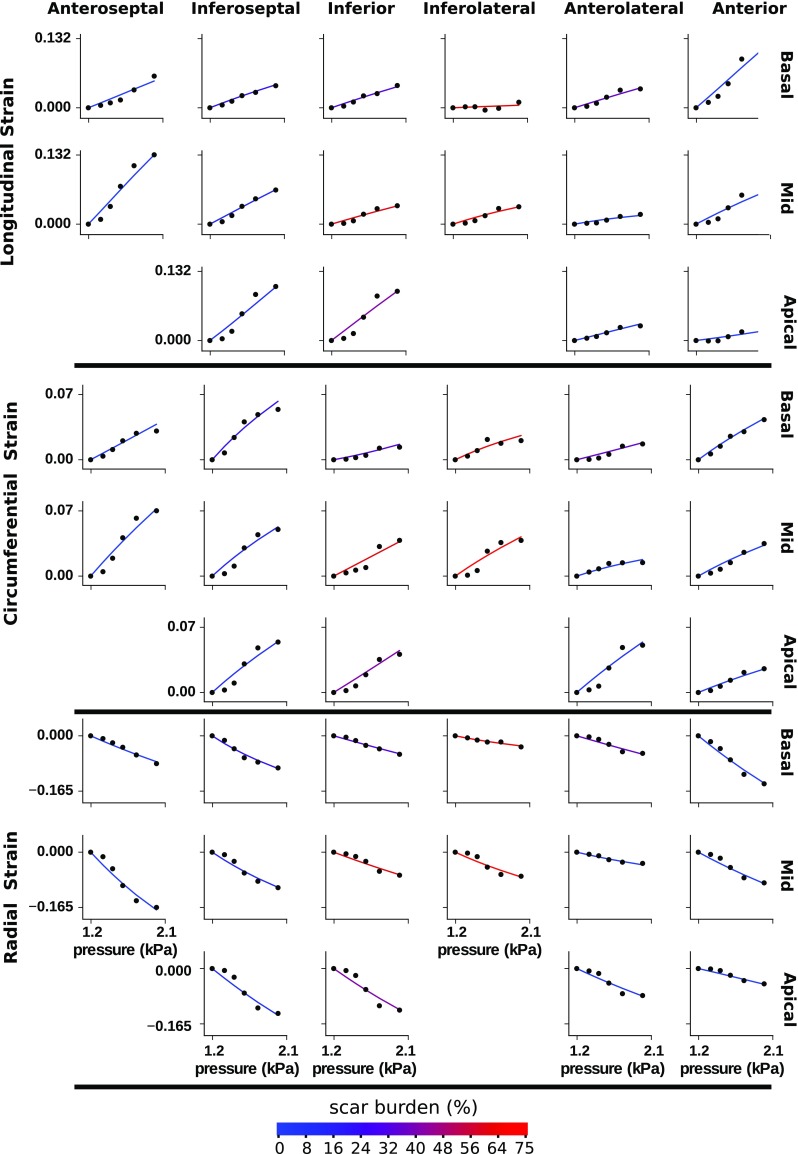
Optimized model (solid line) versus measured (black dot) strain components averaged over the volume of each AHA zone. The reference geometries for the strain measurements are derived from the echo image at 1.2 kPa in the case of the measured strain, and the model at 1.2 kPa in the case of the model derived strain. The line colouring indicates the relative amount of scar in a segment as given by Fig. 3

The best fitting parameter fields (corresponding to the first row of Table 2) are visualized in the top row of Fig. 6. We note that these fields are fairly smooth, yet show large variation across the ventricle. We also compare strain curves generated by the optimized model to the patient strains in Fig. 7.

### Stability of optimized material parameters under mesh refinement

In order to test the effect of mesh refinement on the estimated material parameters, we have carried out a parameter estimation with a slightly finer mesh (1117 vertices, 3373 elements). This estimation was initialized with the same constant values that were used previously in the best fitting optimization to clinical data. The target data were the clinical strains. The resulting optimal material parameter fields are shown in the bottom two rows of Fig. 6. We note that the corresponding original and higher resolution parameters appear to be very similar. We note that the higher resolution parameters came with an increased computational cost, as the time required for an average evaluation of the total functional increased from 30 s to 46 s as compared to the original resolution.

### Regional stress–strain relationships

The personalization of the mechanics model to the patient data resulted in four material parameters fields that were resolved in space over the ventricular geometry. We combined these four parameters into a more intuitive visualization of stiffness by considering regional stress–strain relationships. This allows for regional comparisons to be made for a given level of strain, as stiffer materials give higher stresses given the same strain.

**Fig. 8:**
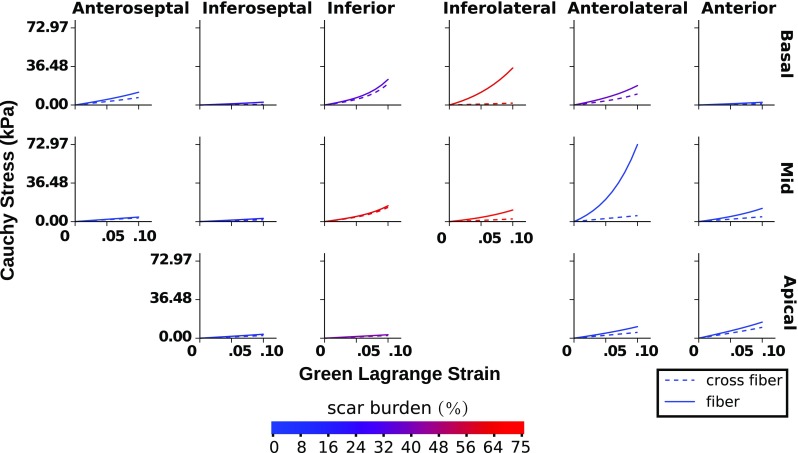
Regional fibre and cross-fibre stress–strain curves generated from simulated equi-biaxial extension tests. In each AHA region the spatial average of the optimal material parameters is used in the simulated extension experiment. The colour of each line indicates the corresponding regional scar burden value

Regional stress–strain curves were calculated with in silico equi-biaxial extension tests, using analytical values for the stresses based on [Eqs. 17, 18 of Holzapfel and Ogden (2008)]. A test was conducted per AHA region using the average of the material parameter fields over the corresponding region. The resulting stress–strain relations along the fibre and cross-fibre directions are presented in Fig. 8.

## Discussion

By applying an adjoint gradient-based data assimilation method, we were able to estimate spatially heterogeneous material properties in an infarcted left ventricle with a good match of simulated to measured strains. This has important implications for the use of computational mechanics models in planning and optimizing therapies in silico. Conditions such as myocardial infarction are local and lead to elastic heterogeneities which should be accounted for in a personalized model. This study presents a general and flexible method to account for these elastic heterogeneities.

Our experiments with synthetic data indicate that fairly accurate reproductions of spatially varying parameters are possible in the absence of noise, as the relative $L^2$ errors were less than 5% for all parameters in this case. In the presence of Gaussian noise the relative errors in the *a*-type parameters increased slightly, but were still below 6%. The effect of the noise on the reproduced *b*-type parameters was more pronounced, and in particular, errors in the $b_f$ parameter for the 0.02 and 0.03 mm noise cases were large enough that the spatial gradient present in the ground truth $b_f$ parameter could no longer be reproduced. These results suggest that spatial heterogeneities can be more robustly estimated with *a*-type parameters rather than *b*-type exponential parameters. Indeed, several recent data assimilation studies have limited parameters estimations to the *a*-type parameters only (Hadjicharalambous et al. 2015; Asner et al. 2017).

We note that the optimal $I_{\text {data}}$ values were two orders of magnitude higher in the clinical case than in the synthetic case. This could be due to higher noise in the clinical data and or modelling error in the representation of in vivo cardiac motion (1). Similarly, the optimal $I_{\text {smooth}}$ values were several orders of magnitude higher in the clinical case than in the synthetic case. This is due to relatively higher gradients in the optimal material parameters fitted to the clinical data.

In both simulated and measured patient data, we noticed that the heavily infarcted region encompassing inferior to inferolateral segments at the base and the mid-posterior segment differed in several ways from healthy segments. In these infarcted segments strains were smaller, and the simulated equi-biaxial stress–strain relationships showed greater fibre stresses. Additionally, the optimal *a* and $a_f$ material parameters are larger in the infarcted anterolateral segment, and there is a band of high *a* parameter value running through the infarcted inferior segments. These observations indicate increased myocardial stiffness. This is consistent with the increased stiffness observed in healing infarcts during an ex vivo tissue experiment (Gupta et al. 1994) and in previous computational modelling with in vivo data (Mojsejenko et al. 2015).

We also observed that the mid-anterolateral segment was identified as free from scar in the late enhancement MRI analysis, yet showed signs of stiffness similar to the heavily infarcted segments described above, that is both low strain and high simulated stress. Such apparent stiffness in a healthy segment is consistent with an infarction impairing the mechanics of neighbouring healthy tissue (Holmes et al. 2005) or could be an effect of myocardial border zone tissue.

Ideally, model material parameters should be uniquely identifiable from in vivo data in order to produce potentially useful biomarkers for clinical practice. Recently, it has been shown that the linear parameters *a* and $a_f$ of a reduced Holzapfel–Ogden law (1), are structurally identifiable (Hadjicharalambous et al. 2015). Structural identifiability means that there exist sets of model loaded states such that only one set of parameters produces them, making it theoretically possible to uniquely identify the parameters. Our in vivo data are corrupted by noise, which makes the question of the unique identifiability of parameters more complex. Additionally, we have optimized the exponential *b* and $b_f$ parameters in our in vivo experiment, for which possible structural identifiability is still unknown. Last but far from least, we have spatially resolved all of the parameters, thereby greatly increasing their dimensionality. Under such circumstances the theoretical identifiability of material parameters is an open question.

To improve the identifiability of material parameters in our estimations, we have added regularization to the optimized functional. Indeed, Fig. 5 confirms the existence of several material parameter sets that fit the model to the data very similarly, but differ in their smoothness. By choosing a corner point in the space of optimized data and smoothness functionals our aim was to pick the smoothest set of elastic parameters that still fit the data well. However, even with the regularization, our parameter estimation still showed a dependency on the choice of initial parameters, and a variety of results were obtained (Table 2). Nevertheless only one parameter set fit the best, allowing us to choose it from among the others.

## Limitations

The identifiability of material parameters was limited in our study, and all optimizations depended upon their initial guess. This dependence is demonstrated in Table 2 by the variety of minima. In the future it would be of interest to further examine constraints to spatially resolved material parameters, ideally yielding an optimization procedure that yields the same parameters regardless of the initialization. One such possible constraint is the left ventricular chamber volume, which has been previously matched together with strain data (Balaban et al. 2017; Mojsejenko et al. 2015; Sun et al. 2009). Further possible constraints are aggregated geometry measures such as LV twist, and long- and short-axis motion. These have been shown to improve identifiability of elastic parameters in experiments with mouse ventricles (Nordbø et al. 2014).

Further limitations were related to the rule-based fibres, mechanics modelling, computational efficiency, and strain and pressure synchronization

### Rule-based fibres

The fibre orientations in our model were generic and not patient specific. As a result, healthy fibre angles were used in infarcted areas. Previous studies have shown that fibre orientations of infarcted areas can be significantly different from healthy tissue (Mojsejenko et al. 2015; Fomovsky et al. 2012). If this effect were incorporated in our parameter estimation, we would expect a change in the optimal material parameters in the infarcted areas, especially in the $a_f$ and $b_f$ parameters, which control the amount of anisotropy in the model along the fibre direction. In the future, further improvements to diffusion-tensor MRI technology may allow for in vivo identification of local myocardial fibre directions, which would allow for the fibre directions to be directly incorporated into the optimized model without needing to be estimated.

### Mechanics modelling

The image-based reference geometry contained a pressure load that was not accounted for in the current study as 0 pressure was assumed for the reference geometry. Using recently developed techniques, it is possible to calculate a pressure-free reference geometry simultaneously with material parameter estimation (Nikou et al. 2016). Applying this technique in our study was unfortunately not possible as the unloaded mesh self-intersected partway into the calculation when we attempted it.

Active tension was assumed to vanish in our model. Typically this tension has decayed to 0 in the diastasis phase of a healthy heart, but may extend into atrial systole under pathological conditions. If active tension were present in the diastasis phase of our patient, then it could add additional stiffness to the myocardium. At the same time, the release of active tension could contribute to strain in atrial systole. Missing these effects would lead to potential overestimation of passive tissue stiffness in the first case and an underestimation in the second.

The computational model lacked several relevant physical effects, notably inertia, viscoelasticity, residual stresses in the unloading geometry, mechanical coupling of the LV to the right ventricle and atria, the effect of sheet microstructure, and tissue compressibility due to blood entering and exiting the ventricle via coronary vessels. The spring constant at the base was a rough approximation and could be replaced by displacement data at the basal boundary if it were available. The apex of the computational model was free, while longitudinal motion at the base was fixed. The in vivo situation is the opposite, the base moves longitudinally, and the apex is mostly stationary.

### Computational efficiency

The spatial discretizations of the material parameters were not optimized. Instead, the computational mesh used to solve the variational equation of motion (4) was also used for the representation of the spatial parameters due to ease of implementation. It is possible that a coarser representation of the material parameters could have also produced good model-data fits. Using fewer parameters could potentially improve the identifiability of parameters and reduce the number of SQP iterations needed to find a minimum.

For the sake of computational efficiency, the resolution of the mesh was not increased to the point of obtaining a numerically convergent solution. Errors in the discretization of the hyperelastic variational equation (4) may have affected the optimized elastic parameter values. However, the results of our test optimization with a finer mesh indicate that any errors due to insufficient mesh resolution did not substantially affect the overall pattern of the optimized parameters.

### Strain and pressure synchronization

LV pressure and strain measurements were not taken simultaneously and had to be synchronized in our study. Though both strain and pressure measurements were taken when the patient was relaxed and prone, there could have been slight differences in heart rate which would confound the strain–pressure synchronization.

## Conclusion

Adjoint-based data assimilation has been used to personalize a mechanics model to reflect the heterogeneity in material properties throughout an infarcted human left ventricle. Further trials with more datasets and more methodological development are warranted in order to evaluate the applicability of the technique.
